# Photo Quiz: Globose Yeast‐Like Forms With Multiple Peripheral Buds in Urine and Cervical Cytology

**DOI:** 10.1002/dc.70200

**Published:** 2026-08-11

**Authors:** Adil Menon, Rachel Koch, Caroline Abramovich

**Affiliations:** ^1^ Department of Pathology Case Western Reserve University Cleveland Ohio USA; ^2^ Department of Pathology MetroHealth Medical Center Cleveland Ohio USA

**Keywords:** cervical cytology, *Cokeromyces recurvatus*, diagnostic pitfall, dimorphic fungus, morphologic mimic, *Paracoccidioides brasiliensis*, photo quiz, urine cytology

## Abstract

A 26‐year‐old immunocompetent woman with a history of noninvasive, low‐grade papillary urothelial carcinoma presented with persistent upper abdominal pain. Urine cytology, performed for bladder tumor surveillance, revealed globose, yeast‐like forms (10–30 μm) with thick walls and multiple narrowly attached peripheral buds in a “ship wheel” configuration—morphology classically associated with *Paracoccidioides brasiliensis*. Three months later, morphologically identical organisms were identified on routine cervical Papanicolaou (Pap) test, confirmed by PAS and GMS stains on cell block material. Neither specimen showed significant inflammation or epithelial abnormality. Clinical and epidemiologic features were inconsistent with paracoccidioidomycosis: the patient was a young woman of reproductive age (male‐to‐female ratio 13–20:1 in chronic paracoccidioidomycosis), had no travel to an endemic region, and lacked pulmonary, mucocutaneous, or systemic disease. The morphologic findings were attributed to *Cokeromyces recurvatus*, a rare dimorphic mucormycete whose sporangiola are virtually indistinguishable from the multiple‐budding yeast cells of *P. brasiliensis* on cytologic preparations. This case is novel in two respects: (1) it represents the first reported simultaneous detection of *C. recurvatus* in both urine and cervical cytology from the same patient, demonstrating multisite genitourinary colonization and (2) identification via urine cytology performed for bladder tumor surveillance extends awareness of this organism beyond the Pap test setting to urinary cytopathology. With fewer than 15 published human cases, this report reinforces that morphology alone is insufficient for definitive fungal identification when an endemic dimorphic fungus is suspected, and that culture or molecular confirmation (broad‐range fungal PCR with DNA sequencing) is essential to avoid misdiagnosis and unnecessary prolonged antifungal therapy.

## 1 Case Presentation

A 26‐year‐old woman with a history of noninvasive, low‐grade papillary urothelial carcinoma presented with persistent upper abdominal pain of several months' duration. The pain was localized to the upper abdomen near the inner rib margin and left of the umbilicus, described as a “pinching” and “sore” sensation, not relieved by acetaminophen and not associated with nausea, vomiting, weight loss, or night sweats.

Urine cytology, performed as part of bladder tumor surveillance, revealed multiple rounded, globose, yeast‐like forms measuring 10–30 μm in diameter with thick walls and multiple narrowly attached peripheral buds arranged in a “ship wheel” configuration (Figures 1, 2, 3, 4). No significant inflammatory background was present. No epithelial atypia was identified.

**FIGURE 1:**
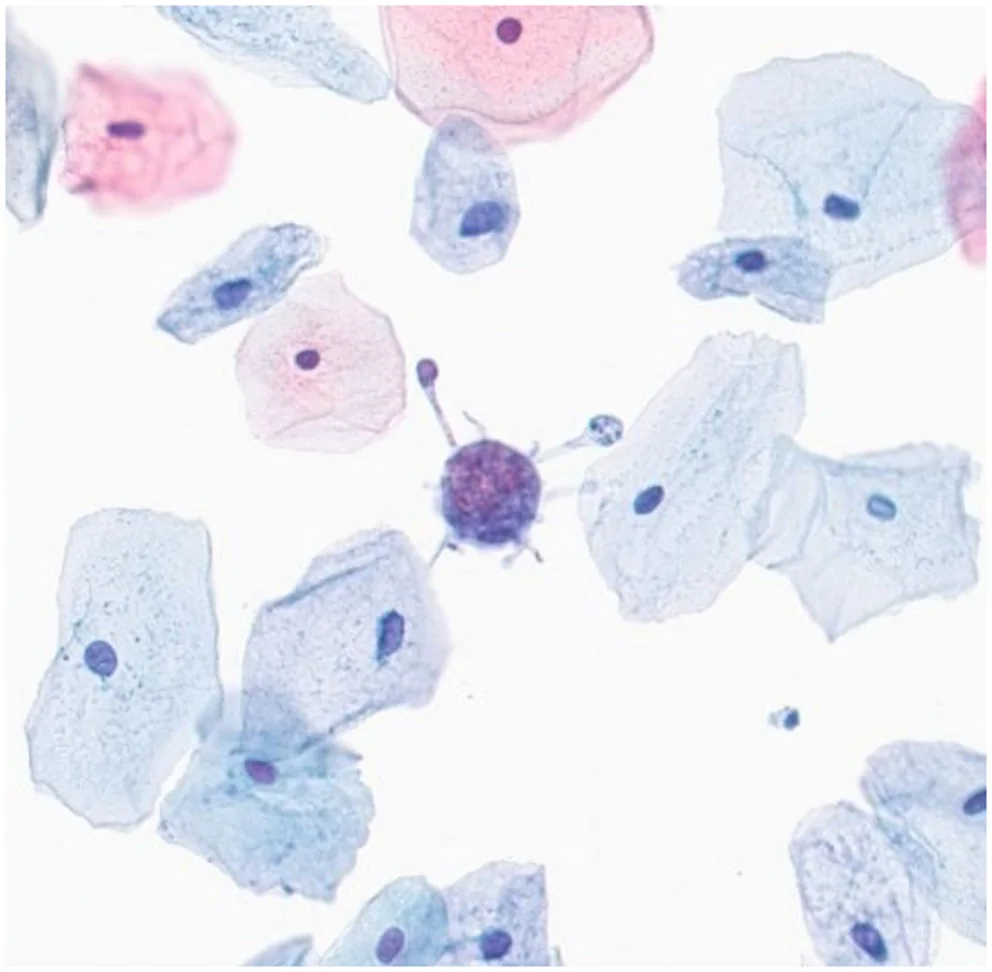
Urine cytology (Papanicolaou stain) showing a single globoid, thick‐walled yeast‐like form with short, narrowly attached peripheral buds radiating from the central body, producing a “mariner's wheel” appearance mimicking Paracoccidioidomycosis. The background consists of mature superficial and intermediate squamous cells without atypia and without an inflammatory reaction.

**FIGURE 2:**
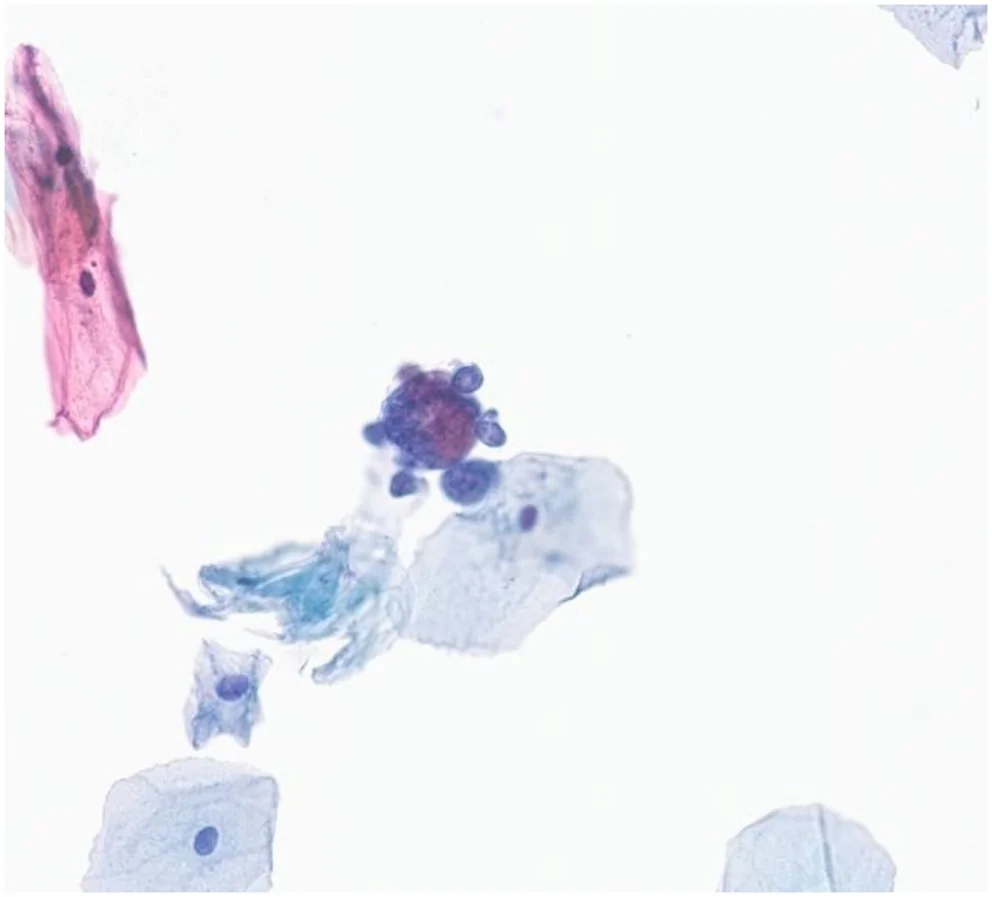
Urine cytology (Papanicolaou stain) displaying a large parent cell bears multiple uniform, narrowly attached daughter buds arising circumferentially; hyphae and pseudohyphae are absent. Adjacent mature squamous cells show no cytologic atypia, and the background is clean and noninflammatory.

**FIGURE 3:**
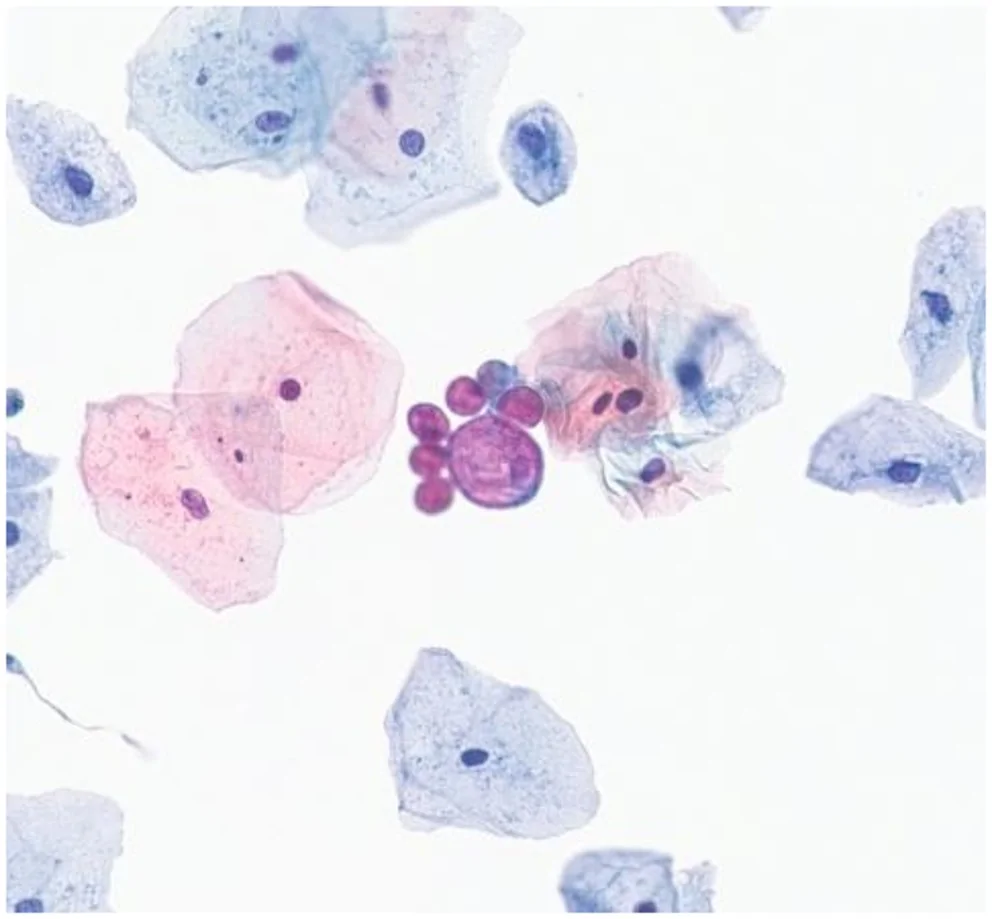
Urine cytology (Papanicolaou stain) showing a cluster of thick‐walled, narrowly attached buds surrounds the central globose body, overlying benign mature squamous cells. No hyphal forms, internal endospores, or associated inflammatory infiltrate are present.

**FIGURE 4:**
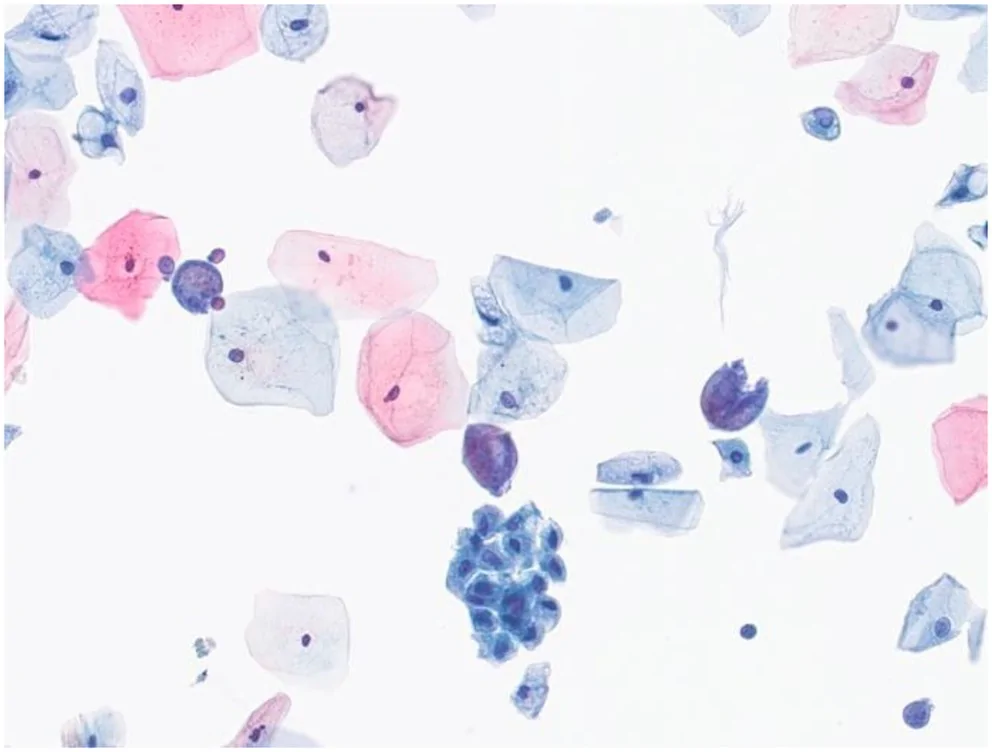
Urine cytology (Papanicolaou stain) displaying budding yeast‐like forms scattered singly among mature squamous cells. The epithelial component is negative for intraepithelial lesion or malignancy, with no acute inflammation.

Three months later, a routine cervical Papanicolaou (Pap) test demonstrated morphologically identical organisms—globose forms with multiple narrow‐based peripheral buds—confirmed by PAS and GMS stains on cell block material. The background was again without epithelial abnormality or significant inflammation.

The patient was immunocompetent with no history of immunosuppressive therapy, HIV infection, or organ transplantation. She had no pulmonary symptoms, mucocutaneous lesions, or lymphadenopathy.

**Questions**

1. What is the most likely morphologic diagnosis based on the cytologic appearance?
2. What is the critical diagnostic pitfall illustrated by this case?
3. What confirmatory testing should be performed?

**Answer to Photo Quiz**

**Diagnosis: *Cokeromyces recurvatus* Mimicking *Paracoccidioides Brasiliensis***

## 2 Discussion

The morphologic appearance of globose, yeast‐like forms (10–30 μm) with multiple narrowly attached peripheral buds in a “ship wheel” or “mariner's wheel” pattern is classically associated with *P. brasiliensis*, the agent of paracoccidioidomycosis [1, 2]. However, this case illustrates a well‐documented but under‐recognized diagnostic pitfall: *C. recurvatus*, a rare dimorphic mucormycete (order Mucorales), produces sporangiola—small, spherical reproductive structures borne on recurved sporangiophores—that are morphologically virtually indistinguishable from the multiple‐budding yeast cells of *P. brasiliensis* in cytologic preparations [3, 4].

In this patient, the initial morphologic interpretation of both the urine and cervical cytology specimens was “consistent with Paracoccidioides species.” However, several clinical and epidemiologic features were inconsistent with true paracoccidioidomycosis:

- Clinical paracoccidioidomycosis is strikingly rare in women of reproductive age (male‐to‐female ratio of 13:1–20:1 in the chronic adult form), attributed to estradiol‐mediated inhibition of the mycelial‐to‐yeast transition via a specific cytosol binding protein in the fungus [5].
- The patient had no travel to or residence in a Paracoccidioides‐endemic region (Latin America).
- There were no pulmonary symptoms, mucocutaneous lesions, lymphadenopathy, or adrenal insufficiency—the typical manifestations of paracoccidioidomycosis [6].
- Imaging was entirely normal.
- The absence of significant inflammation or tissue reaction in both specimens was more consistent with colonization than infection.

This pitfall has been documented in multiple prior reports. Odronic et al. described two cases of *C. recurvatus* in liquid‐based Pap tests from healthy, asymptomatic women (ages 26 and 47) in which the morphology was initially interpreted as consistent with *P. brasiliensis* ; broad‐range fungal PCR and DNA sequencing showed an exact match for *C. recurvatus* [7]. Paquette et al. reported *C. recurvatus* in a Pap test from an asymptomatic pregnant patient, noting that “rare fungi were considered including *Paracoccidioides brasiliensis* due to the ‘mariner's wheel’ morphology” before culture confirmed *C. recurvatus* [8]. Nielsen et al. reported *C. recurvatus* from peritoneal fluid in a cat, initially misidentified as *Coccidioides immitis*, and noted that the tissue form of *C. recurvatus* is “morphologically similar to those of *Coccidioides immitis* and *Paracoccidioides brasiliensis* and may be misdiagnosed as one of these organisms on the basis of cytologic or histopathologic specimens” [4]. Kemna et al. described *C. recurvatus* isolated from endocervical specimens in a diabetic patient, with fungus‐like structures observed in Papanicolaou‐stained cervical smears prepared 1 year apart [9].

Notably, Odronic et al. suggested that two prior case reports describing *P. brasiliensis* in cervical Pap tests—including the report by Alsharif et al. of “ship's‐wheel” morphology in a liquid‐based Pap test from a healthy 29‐year‐old pregnant woman diagnosed as “consistent with *Paracoccidioides*” without culture or molecular confirmation—may have been, in fact, misdiagnoses of *C. recurvatus* [7]. This hypothesis underscores the critical importance of molecular or culture‐based confirmation when morphology alone suggests an endemic dimorphic fungus in the female genital tract.

The present case adds to this literature in two respects. First, it represents the first reported instance of *C. recurvatus* detected simultaneously in both urine and cervical cytology from the same patient, suggesting colonization of the genitourinary tract at multiple sites. Second, identification through urine cytology performed for bladder tumor surveillance represents a new clinical context for this organism, extending awareness beyond the Pap test setting to urinary cytopathology. As of the most recent published review, only 14 human cases of *C. recurvatus* had been reported, with 8 (57%) involving the female genital tract [3].

### 2.1 Clinical Significance of *C. recurvatus*

In the female genital tract, *C. recurvatus* has consistently been reported as a nonpathogenic colonizer in immunocompetent patients, with no associated inflammation or epithelial abnormality [3, 4]. However, invasive disease has been reported in immunocompromised patients: severe diarrhea in a bone marrow transplant recipient and isolation from pleural and peritoneal fluid in a patient with ruptured duodenal ulcer [10, 11]. No antifungal treatment is indicated for genital tract colonization in immunocompetent patients.

### 2.2 Consequences of Misdiagnosis

Misidentification as *P. brasiliensis* could lead to unnecessary, prolonged antifungal therapy with associated hepatotoxicity, drug interactions, cost, and patient anxiety [12].

## 3 Learning Points

1. Morphology alone is insufficient for definitive fungal identification. A 10‐year retrospective review found a 21% discrepancy rate between morphologic identification and culture results, including misidentification of yeast forms. Culture or molecular confirmation (broad‐range fungal PCR with DNA sequencing) is essential when an unusual or endemic dimorphic fungus is suspected on cytology [13].
2.
*C. recurvatus* sporangiola are the most important morphologic mimic of *P. brasiliensis* multiple‐budding yeast cells. Both produce globose forms (10–30 μm) with multiple narrowly attached peripheral structures in a “ship wheel” pattern. This mimicry has been documented in at least four published reports [3, 4, 6, 7, 8].
3. Clinical and epidemiologic context must inform morphologic interpretation. Paracoccidioidomycosis in a young woman of reproductive age, outside an endemic region, without pulmonary or systemic disease, should prompt strong consideration of alternative diagnoses.
4.
*C. recurvatus* can also mimic *C. immitis*. In histologic sections, the sporangiola‐filled sporangia of *C. recurvatus* can resemble the endospore‐filled spherules of Coccidioides [6].
5. The female genital tract is the most reported site for *C. recurvatus* in humans. Awareness of this organism is, particularly, important for cytopathologists interpreting Pap tests. Prior reports of *P. brasiliensis* in cervical Pap tests without molecular or culture confirmation may represent *C. recurvatus* misdiagnoses [3, 7].
6. In immunocompetent patients, genital tract *C. recurvatus* is a colonizer requiring no treatment—in contrast to *P. brasiliensis* , which requires 9–12 months of antifungal therapy. Correct identification prevents unnecessary treatment.
7. When morphology suggests an endemic dimorphic fungus, the following confirmatory workup is recommended: (a) culture at 25°C–30°C for mold phase identification; (b) broad‐range fungal PCR with DNA sequencing; (c) organism‐specific serology (immunodiffusion for *Paracoccidioides*); and (d) clinical‐epidemiologic correlation.

## Funding

The authors have nothing to report.

## Conflicts of Interest

The authors declare no conflicts of interest.

## Data Availability

Data sharing not applicable to this article as no datasets were generated or analysed during the current study.
